# Impact of Fixed-Dose Combination of Germacrone, Curdione, and Furanodiene on Breast Cancer Cell Proliferation

**Published:** 2013-07-02

**Authors:** Qi Kong, Fangyun Sun, Xiuping Chen

**Affiliations:** 1 Department of Laboratory Animal Sciences, Peking Union Medical College (PUMC) and Chinese Academy of Medical Sciences (CAMS), Beijing, China; 2 Department of Pharmacology, Tibet Nationalities Institute, Xianyang, China; 3 State Key Laboratory of Quality Research in Chinese Medicine, Institute of Chinese Medical Sciences, University of Macau, Macao, China

**Keywords:** Fixed-Dose-Combination, Proliferation, Breast Cancer, Intra-Herbal Drug Interactions, Chinese Medicine

## Abstract

**Objective:**

Herb combination has been very popular in traditional medical prescriptions such as Traditional Chinese Medicine (TCM). Persistent efforts and attempts have been made to dissect the action mode of TCM in recent years, which has provided certain evidence for inter-herbal interactions. However, the interactions among different components in a single herb have been largely neglected.

**Materials and Methods:**

In this experimental study, the interactions among different components of a single herb were explored. The effect of three main sesquiterpenes (germacrone, curdione, furanodiene) isolated from *Curcuma WenyujinY.H.Chenet C Ling* on MDA-MB-231 and MCF-7 breast cancer cell proliferation alone or in combination with a fixed-dose-combination was investigated.

**Results:**

Furanodiene significantly inhibited cancer cell proliferation while germacrone and curdione showed no effect. Germacrone enhanced furanodiene’s anti-proliferative effect. Curdione showed no effect on furanodiene’s anti-proliferative effect but partly reversed the anti-proliferative effect of germacrone and furanodiene combined. The morphological and mitochondrial membrane potential (*Δψm*) changes showed similar results. However, they demonstrated complicated interactions on the expression of apoptotic-related proteins and key signal transduction proteins.

**Conclusion:**

Unpredictable and complex interactions among different components in *Curcuma WenyujinY.H.Chenet C Ling* may exist. The intra-herb interactions should be taken into consideration when attempts are made to interpret the art of TCM formulation or other similar recipes.

## Introduction

Accumulated data have demonstrated that complementary and alternative medicine (CAM) shows beneficial effect in the treatment of several kinds of cancers (1-3). Traditional Chinese medicine (TCM), an empirical system with more than 2500 years history of application, has been considered as one of the typical representatives of CAM by some researchers. In fact, TCM has been the mainstream medicine system in the long history of China and has been widely accepted by most Chinese even to this date. The basic therapeutic unit in TCM is medicinal herb, which mainly includes thousands of medicinal plants. The most popular treatment form of TCM is herbal formula (Fu-Fang), which is usually grouped by two or more medicinal herbs. It is generally accepted that there are complicated interactions among different herbs and some constructive approaches, such as systems biology and network pharmacology have been tried to assess the usefulness and dissect the mechanisms of TCM (4-6).

According to the TCM theory, the herb combination application could amplify the therapeutic efficacies of each herb and minimize the adverse effects. Considering the complicated components in a certain formula, many researchers and practitioners believed that, at least in some formulae, multiple components could hit multiple targets and exert synergistic therapeutic efficacies. With the ambitious plan of TCM modernization in China, more and more studies have been performed to investigate the interaction of different herbs in TCM formulae. A recent study dissected the mechanisms of a formula termed realgar-indigo naturalis in an acute promyelocytic leukemia (APL) model by investigating the interaction of three pure compounds namely tetraarsenictetrasulfide, indirubin, and tanshinone IIA which were selected to represent three herbs respectively: realgar, indigo naturalis, and *salvia miltiorrhiza*. Results showed that the combination yields synergy in the treatment of APL (7). Furthermore, the interaction of herb-herb and herbdrug has been a well-documented research interest in recent years (8-11). However, the philosophy be hind these studies has been frequently questioned because the complicated components in each herb were simplified to a single compound and the intraherbal interactions have been largely neglected.

**Fig 1:**
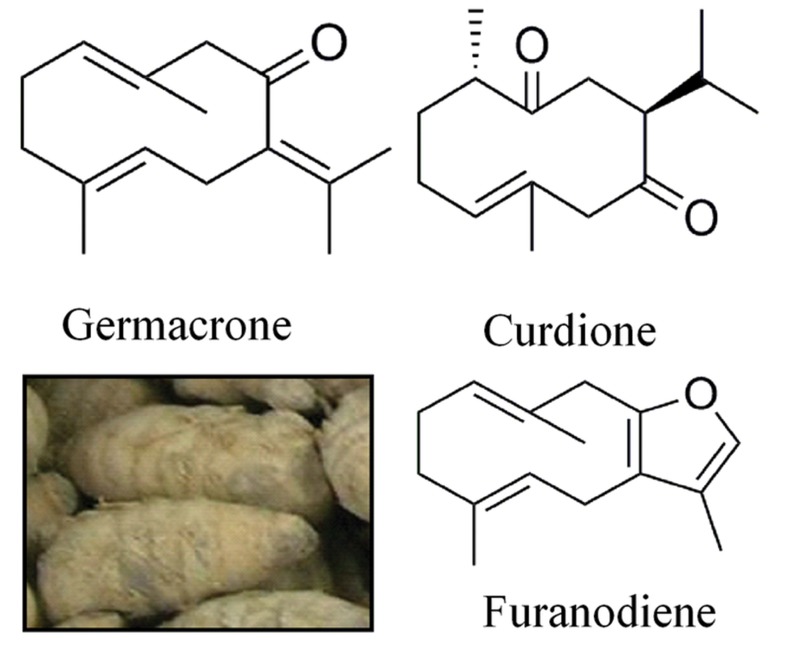
Curcuma WenyujinY.H. Chen et C Ling (The lower left corner) and chemical structure of germacrone, curdione, and furanodiene.

*Curcuma WenyujinY.H.Chenet C Ling* Ling is a commonly prescribed Chinese herb with anti-cancer potentials (12). The sesquiterpenes have been identified as its main bioactive components with anti-cancer effect both *in vitro* and *in vivo* (13-16). A previous study identified that germacrone, curdione, and furanodiene were the three main components in the herb with an approximate molar ratio of 1:3:3 (17) (Fig 1). Also, germacrone and furanodiene have been chosen as the index ingredients for *Curcuma WenyujinY.H.Chenet C Ling* quality control. In view of their high content, structural similarities and reported activities, there maybe interactions among them. Therefore, in present study, their interactions are determined in an anti-proliferation model on breast cancer cells with a fixed-dose-combination.

## Materials and Methods

### Reagents

In this experimental study, germacrone, curdione, and furanodiene used in this experimental study were purchased from the National Instisutes for Food and Drug Control (Beijing, China). The RPMI-1640 culture medium was obtained from Gibco (USA). Fetal bovine serum (FBS), phosphate-buffered saline (PBS), penicillinstreptomycin (PS), and 0.25% (w/v) trypsin/1mM EDTA were purchased from Invitrogen (USA). 3-[4,5-dimethyl-2-thiazolyl]-2,5-diphenyl tetrazolium bromide (MTT), 5, 5', 6, 6'- tetrachloro-1, 1', 3, 3'-tetraethyl-benzimidazolylcarbocyanine iodide (JC-1), phenylmethanesulfonyl fluoride (PMSF) andprotease inhibitor cocktail were purchased from Molecular Probes (USA). RIPA lysis buffer was obtained from Santa Cruz (USA). Primary antibodies against Bcl-2, p-Bcl-2, Bclxl, Bax, Bad, Bok, Bim, caspase-9, cleaved caspase- 9, PARP, NF-κB, p38MAPK, p42/44MAPK, β-actin, and secondary antibodies were obtained from Cell Signaling (USA). In this paper, A stands for germacrone (14.3 μM), B stands for curdione (42.9 μM), C stands for furanodiene (42.9 μM), AB stands for A (14.3 μM) and B (42.9 μM) combined while ABC is A (14.3 μM), B (42.9 μM) and C (42.9 μM) combined together and the rest follows the same pattern.

### Cell culture

Human breast cancer cell lines, MCF-7 and MDA-MB-231 were obtained from ATCC (USA). Cells were cultured in medium containing RPMI- 1640, antibiotics (100 U/mL penicillin, 100 μg/mL streptomycin), and 10 % (v/v) heat-inactivated FBS at 37˚C under 5 % CO_2_.

### Morphological observations

Exponentially growing MCF-7 (1.5×10^4^) cells in 100 μL medium were seeded in 96-well plates and treated with A (14.3 μM), B (42.9 μM) and C (42.9 μM) alone or in a combination for 24 hours. The cellular morphology was observed with AxioCam HRC CCD camera (Carl Zeiss).

### MTT assay

The cells were cultured and treated with A, B and C alone or in a combination as described above. The cell viability was determined by the MTT assay as previously described (18).

### JC-1 assay

MDA-MB-231 cells were cultured and treated with A, B and C alone or in a combination for 4 hours. The mitochondrial membrane potential (*Δψm*) was monitored by JC-1 staining as previously described (19).

### Western blot assay

To determine the effect of A, B and C alone or in a combination on protein expression, MDA-MB-231 cells were treated for 24 hours. The expression of apoptotic-related proteins and key signaling transduction proteins were determined by Western blotting as previously reported (20).

### Data analysis

The MTT data were presented as mean ± SD. The significance of intergroup differences was calculated by one-way analyses of variance (one-way-ANOVA) using SPSS 11.5 software. Statistical differences were considered significant at p<0.05.

## Results

### The effect of A, B and C alone or in a combination on breast cancer cell proliferation

**Fig 2:**
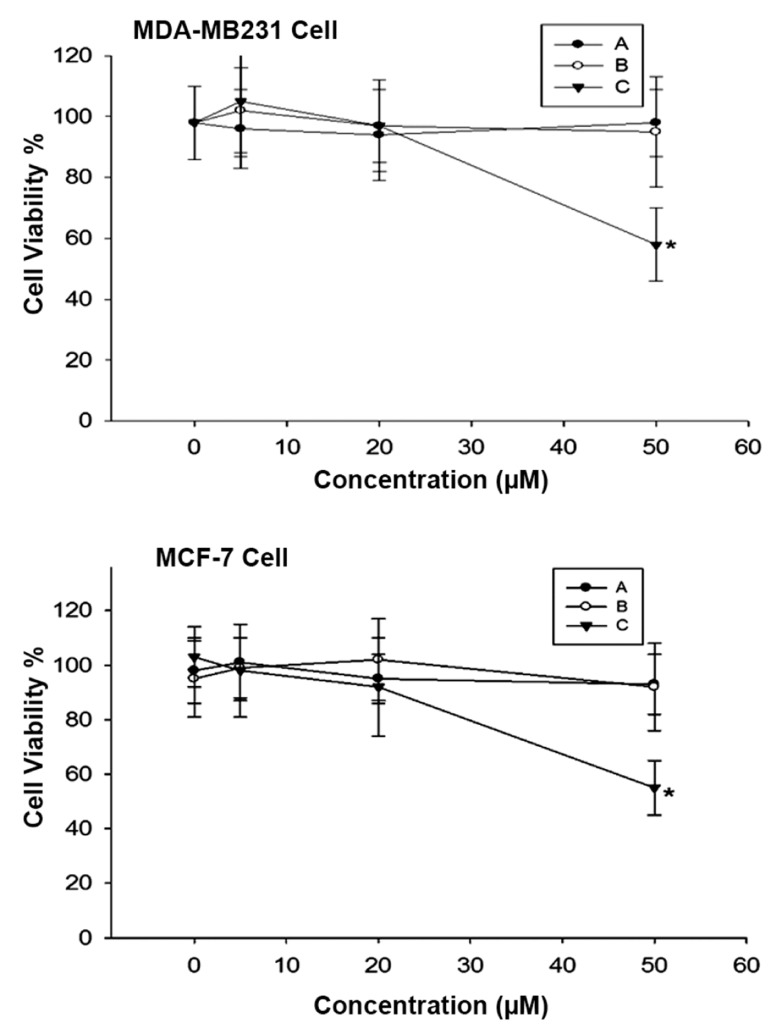
Effect of germacrone (A), curdione (B) and furanodiene (C) (0-50 μM) on MCF-7 and MDA-MB-231 cell proliferation. *; P<0.05 vs. 0 μM.

Firstly, the effect of A, B and C alone on MDAMB- 231 and MCF-7 cell proliferation was determined. As shown in figure 2, neither A nor B showed cytotoxic effect on both cell lines at 50 μM while C dramatically inhibited both cell line proliferation.

**Fig 3:**
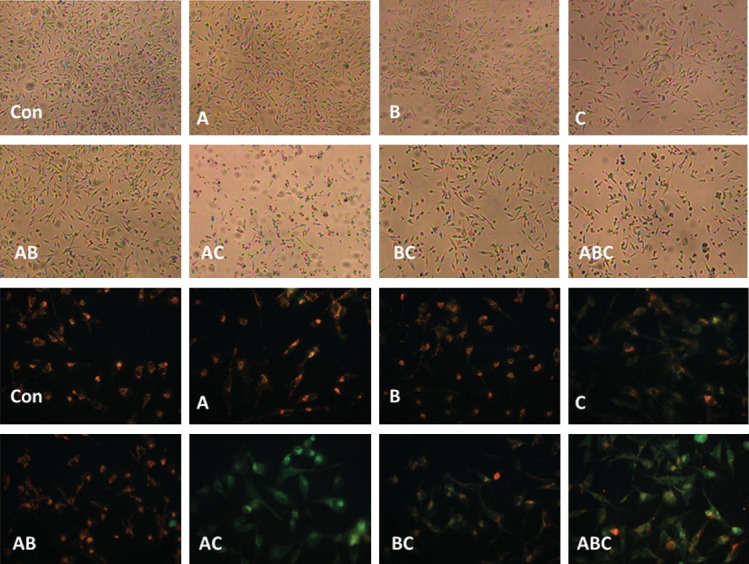
Morphological changes of MDA-MB-231 cells after 24 hours of germacrone (A), curdione (B) and furanodiene (C) alone or in combination treatment (up) and JC-1 staining (down). After A and B treatment, there is no obvious morphological changes in the cells. C treated group showed decreased cell number. However, after combination of these compounds, the cell numbers decreased and the cells became round. Some dead cells were also observed in C, AC, BC and ABC treated groups. The red fluorescence suggested that the mitochondrial membrane potential (*Δψm*) was high while the green fluorescence suggested that the *Δψm* was low. The magnification for morphological study and JC-1 staining study were ×20 and ×40 respectively.

**Fig 4:**
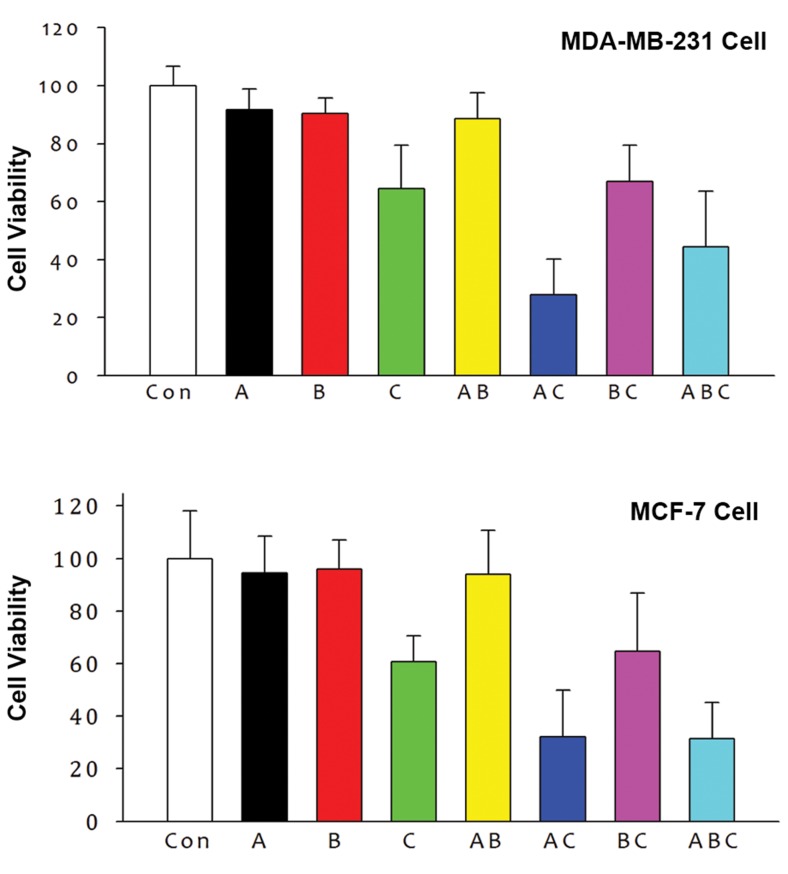
Effect of germacrone (A), curdione (B) and furanodiene (C) alone or in a combination on cell proliferation of MDA-MB-231 and MCF-7 breast cancer cells.

No obvious morphologic changes were observed after treating with A and B alone or in combination while C significantly changed the cell morphology and decreased the cell number. AC, BC, and ABC induced obvious morphologic changes (Fig 3). The cell viabilities after treatment are shown in figure 4. The statistical differences among every two groups were calculated as shown in table 1. The p values in table 1 indicated that A, B, and AB showed no effect on cell proliferation. C inhibited cell proliferation by 40%, which was increased to 70% after combined with A (Fig 4). However, there was no significant difference between B and BC on viability in both cell lines. Furthermore, statistically significant differences were also observed between AB, AC and BC with ABC in MDA-MB-231 cells.

**Table 1:** Pairwise statistical differences among groups after germacrone (A), curdione (B), and furanodiene (C) alone or combined treatment in MDA-MB-231 cells


	Con	AB	AC	BC	ABC

**A**	P1	P2	P3		P4
**B**	P5	P6		P7	P8
**C**	P9		P10	P11	P12
**AB**	P13		P14	P15	P16
**AC**	P17	P18		P19	P20
**BC**	P21				P22


P >0.05; P1, P2, P5, P6, P11, P13, p<0.05; P4, P8, P10, P12, P15, P16, P19, P20, P21, P22 and p<0.01;P3, P7, P9, P14, P17, P18. Con; Control.

### The effect of A, B and C alone or in a combination on MDA-MB-231 cell Δψm

The effect of A, B and C alone or in a combination on MDA-MB-231 cell *Δψm* is shown in figure 3 (below), which is quite similar to those of MTT results. Compared with the control group, no obvious changes in the red fluorescence were observed in A, B and AB treated group. However, C, AC, BC, and ABC treated groups exhibited increased green fluorescence. Furthermore, more green fluorescence was observed in AC group than that of ABC group. These results showed that *Δψm* decreased in C, AC, BC, and ABC groups.

### The effect of A, B and C alone or in a combination on apoptotic-related protein expression

**Fig 5:**
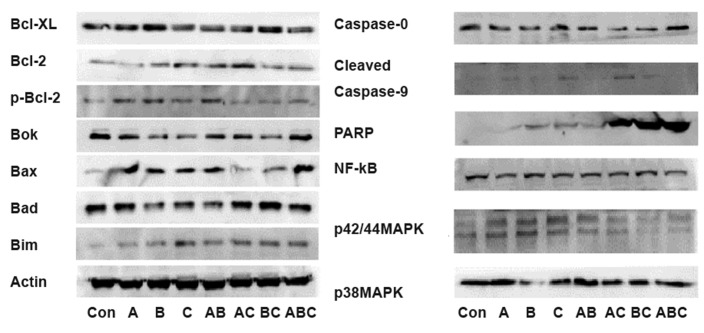
Effect of germacrone (A), curdione (B) and furanodiene (C) alone or combined treatment on the expression of apoptotic-related and key molecular signaling proteins.

Both A and B showed no effect on Bcl-xl, Bcl-2 and Bim expression, increased Bax andp-Bcl-2, and decreased Bok expression. B decreased Bad expression while A had no effect. C increased Bcl-2, Bax and Bim expression and decreased Bcl-xl, Bok and Bad without affecting p-Bcl-2. AB decreased Bcl-xl, Bok and Bad expression, while increasing Bcl-2, p- Bcl-2, Bax, and Bim expression. AC decreased Bclxl, Bok and Bax expression, increased Bcl-2 and Bim without affecting p-Bcl-2 and Bad expression. BC decreased Bok expression, increased Bax and- Bim expression while showing no effect on Bcl-xl, Bcl-2, p-Bcl-2 and Bad expression. ABC decreased Bcl-xl, increased Bax and Bim without affecting Bcl-2, p-Bcl-2, Bok, and Bad expression (Fig 5).

AC and BC decreased caspase-9 expression while C and AC increased cleaved caspase-9 expression. It is interesting to note that B, C, AB, AC, BC ABC could significantly increase PARP expression, especially in AC, BC and ABC groups (Fig 5).

### The effect of A, B and C alone or in a combination on key signaling molecular protein expression

A, B and C alone or in a combination showed no effect on NF-κB expression. It is interesting to note that A, B, C alone increased p42/44 MAPK expression while in a combination showed no effect. A, C, AB, AC, BC, ABC showed no effect on p38 MAPK expression while B decreased p38MAPK expression (Fig 5).

## Discussion

Drug combination therapy, in which two or more agents interact with multiple targets simultaneously, is considered a rational and efficient form of pharmacotherapy designed to control complex diseases such as cancer (21). The fixed-dose combinations are becoming increasingly important andbeing used in the treatment of quite a few diseases such as acquired immune deficiency syndrome (*AIDS*), malaria and tuberculosis. Mixtures of compounds produced by plants may provide important combination therapies and provide clinical efficacy beyond the reach of single compoundbased drugs (22). The application of herb combination based formula in TCM shares a similar therapeutic philosophy with modern drug combination. Therefore, in this regard, many researchers believe that the TCM may have the potential of addressing a relationship between multiple components and drug synergistic effects (23). Furthermore, to interpret the complicated system, systems biology and its approaches have been employed to shed light on the mystery of TCM (4, 5). However, most of these models and studies were documented for constituents within a total extract of a single herb, different herb extracts, as well as between different herbs in a formula (24-27). Little is known about the interactions of different components from a single herb. A, B, and C are three main sesquiterpenes in *Curcumaewenyujin* (17). The MTT assay showed that A hadno effect on the proliferation of both MCF-7 and MDA-MB-231 cell linesbut significantly increased the effect of C on these cells suggesting that A has an enhancing effect on C. However, B did not show such an effect on C. Also, the significant difference between AC and ABC suggested that B could partly reverse the effect of AC though B itself demonstrated no effect on cell proliferation. Thus, the anti-tumor potential of *Curcumaewenyujin* might be underestimated if Aor B was chosen as the representative component. Although both MDA-MB-231 and MCF-7 are breast cancer cell lines, the former is an aggressive and estrogen receptor (ER)-negative cell line, while the latter is an ER positive cell line. MDA-MB-231 cells also demonstrated drug resistant characteristics. Present results suggest that although both cell lines respond equally to these compounds, the underlying mechanisms maybe quite different, which needs further study to elucidate.

Morphological observations demonstrated similar results. JC-1 staining is a common method to study the *Δψm*, an early marker for apoptosis. A and B alone or in a combination did not affect *Δψm* while C significantly decreased *Δψm* suggesting that C might induce apoptosis. This was consistent with previous observations in HL-60 cells (13). Our recent studies also found that A and an extract with A and C as main components inhibited the proliferation of breast cancer cell lines by inducing cell cycle arrest and promoting apoptosis and dose-dependent decrease of *Δψm* (15, 18). The concentration used in the present study is much lower and thus showed no effect on both *Δψm* and proliferation. Actually, our study showed that less than 50 μM A exhibit no significant cytotoxic effect on breast cancer cells (15). Although there are some reports about the bioactivity of B (28, 29), no obvious effect on breast cancer proliferation was observed at the concentration tested in the present study.

To further confirm the interactions among A, B and C at the molecular level, some apoptotic-related proteins and several key signaling molecular were determined. Results demonstrated that A, B and C alone showed complex effects on the expression of Bcl-2 family proteins. For example, C increased the anti-apoptotic protein Bcl-2 and the pro-apoptotic protein Bax, Bim at the same time. Also, it decreased Bok but increased the Bim protein, two other pro-apoptotic Bcl-2 proteins (30, 31). Their combinatorial effect is more complicated as indicated by their effect on the expression of Bax. Combinatorial effect on downstream proteins such as caspase-9 and PARP suggested that they might induce apoptosis. Similar intricate interactions were also observed in the expression of p42/44MAPK and p38MAPK proteins. It should be noted that the cellular results (MTT, JC-1 and morphological changes) showed "homogeneity" while the molecular outcomes (expression of the mentioned proteins) demonstrated "heterogeneity". This divergence could be a reflection of holistic view and analytic reductionism at cellular and molecular level respectively and suggests that it is inappropriate to simplify one herb to a single compound in formula studies.

## Conclusion

Taken together, the present study provides evidence that there are complicated interactions among the three components of *Curcuma Wenyujin*Y.H. *Chenet C Ling* namely germacrone, curdione, and furanodiene. Therefore, intra-herb drug interactions should be taken into consideration when attempts are made to interpret the art of traditional medicines.
